# Out of Nucleus: Serine 727 Phosphorylation Orchestrates Non-Canonical STAT3 Functions—Relevance to Triple-Negative Breast Cancer

**DOI:** 10.3390/ijms27052242

**Published:** 2026-02-27

**Authors:** Daniele Viavattene, Andrea Roberto Marchetti, Nicole Schael, Valeria Poli

**Affiliations:** Department of Molecular Biotechnology and Health Sciences, Molecular Biotechnology Center, University of Turin, Via Nizza 52, 10126 Turin, Italy; daniele.viavattene@unito.it (D.V.); andrearoberto.marchetti@unito.it (A.R.M.); nicole.schael@edu.unito.it (N.S.)

**Keywords:** STAT3, S727, Y705, cancer, breast cancer, Triple-Negative Breast Cancer

## Abstract

Signal transducer and activator of transcription 3 (STAT3) is a central oncogenic hub in several tumors including the Triple-Negative Breast Cancer (TNBC) subtype, where its constitutive activity supports proliferation, metabolic flexibility, tumor progression, immune evasion, and therapeutic resistance. Therapeutic development has largely focused on canonical STAT3 activation driven by tyrosine 705 phosphorylation (p-Y705), which enables dimerization and transcriptional programs. However, accumulating evidence indicates that phosphorylation at serine 727 (p-S727) defines a functionally distinct STAT3 axis, underpinning non-canonical activities across extranuclear compartments that include mitochondria and endoplasmic reticulum/mitochondria-associated membranes. In TNBC, p-S727 STAT3 is frequently prevalent and may sustain oncogenic signaling when p-Y705 is low or pharmacologically suppressed, contributing to metabolic rewiring, redox control, apoptosis resistance, and metastatic fitness. Here, we review the mechanistic basis and clinical correlations of STAT3 p-S727 across cancers with emphasis on TNBC, and discuss how compartmentalized STAT3 pools may integrate kinase signaling, nutrient sensing, and stress responses. We also summarize emerging therapeutic strategies that modulate p-S727—often in conjunction with p-Y705—highlighting proof-of-concept for dual targeting or specific p-S727 to overcome limitations of Y705-centric approaches. Finally, we propose that integrating p-S727/p-Y705 distribution and activity into patient stratification could improve the efficacy–toxicity balance of STAT3-directed therapies in TNBC.

## 1. Introduction

Signal transducer and activator of transcription 3 (STAT3) is a pleiotropic transcription factor that integrates cytokine and growth factor signals to regulate gene expression programs controlling cell proliferation, survival, differentiation, and immune regulation, thus functioning as a central integrator of inflammatory and oncogenic cues [1,2]. Canonical STAT3 activation occurs downstream of cytokine receptors, notably those of the interleukin 6 (IL-6) family, through Janus kinases and non-receptor tyrosine kinases, leading to phosphorylation at tyrosine 705 (Y705, p-Y). This promotes dimerization through reciprocal SH2-pY domains interaction, nuclear accumulation, and binding to γ-activated sequence (GAS) motifs to regulate transcription of STAT3 target genes [1,2,3,4]. Under physiological conditions, this signaling is transient and tightly controlled by negative regulators, including suppressor of cytokine signaling (SOCS) proteins, protein inhibitor of activated STAT3 (PIAS3), and protein tyrosine phosphatases (PTPs), ensuring timely signal termination and preventing uncontrolled gene expression [5,6,7,8].

Despite sparse cases where STAT3 activating somatic mutations have been detected in cancer, in most instances constitutive activation occurs in the presence of the wild type protein [9]. Accordingly, persistent p-Y705 can be driven by chronic stimulation from tumor- and stroma-derived cytokines (e.g., IL-6, IL-11, OSM), aberrant activation of receptor tyrosine kinases such as Epidermal Growth Factor Receptor (EGFR) and Human Epidermal Growth Factor Receptor 2 (HER2), non-receptor tyrosine kinases including Src and Abl, and deregulated G protein–coupled receptor (GPCR) pathways [8,10]. Loss or functional impairment of inhibitory molecules such as SOCS1/3, PIAS3, SHP-1/2, or PTPN2 further stabilize STAT3 activation prolonging its nuclear residency [5,8]. These alterations collectively rewire STAT3 from a transient inflammatory responder to a chronically active oncogenic driver, a state documented across multiple solid tumors and hematologic malignancies [8,10]. Of note, STAT3 activity can contribute to malignant transformation in primary cells, supporting its role as a bona fide oncogenic driver [10,11,12].

However, multiple independent lines of evidence indicate that STAT3-dependent phenotypes cannot be fully accounted for by the canonical transcriptional model. Across diverse systems, genetic ablation of Y705 phosphorylation or disruption of STAT3 DNA binding fails to completely suppress STAT3-driven effects on cellular metabolism, stress tolerance, or survival [13,14,15]. These observations have contributed to the concept of non-canonical STAT3 signaling, encompassing STAT3 functions that are not strictly dependent on Y705-driven nuclear transcription [16,17,18]. Non-canonical STAT3 activities are closely related to its ability to engage distinct subcellular compartments. Beyond its p-Y-dependent nuclear concentration and the diffuse distribution of the non-phosphorylated form, STAT3 also exhibits specific and functional compartmentalization within mitochondria and endoplasmic reticulum (ER)-associated compartments, including mitochondria-associated ER membranes (MAMs) [17,18,19,20].

A key functional determinant of these non-nuclear STAT3 activities is phosphorylation at serine 727 (S727, p-S) [17,18,19,21]. Historically, p-S727 was viewed primarily as an accessory modification that enhances the transcriptional efficiency of Y705-phosphorylated STAT3, e.g., by facilitating recruitment of co-activators and supporting productive transcriptional elongation at a subset of promoters [14,22,23]. Later on, Yang and co-authors showed that p-S727 could negatively regulate STAT3 transcriptional activities by regulating its chromatin binding dwell time [24], suggesting non-linear and context-dependent interplay between the two phosphorylated forms. Accordingly, while germline STAT3 gene inactivation is embryonic lethal, mice carrying a S727A homozygous mutation were normal and viable [23].

More recent evidence supports a broader model in which S727 phosphorylation modulates STAT3 specific activities in diverse subcellular compartments. Indeed, S727 phosphorylation, independently from DNA binding or Y705 phosphorylation, was shown to be required both for mitochondrial STAT3 activities and for its participation in ER-associated signaling complexes [17,19,21,25]. In this framework, S727 is positioned downstream of pathways such as MAPK and mTOR and links growth factor signaling, nutrient sensing, and cellular stress to STAT3-dependent outputs in metabolism, survival, and cellular fitness [13,15,26,27,28].

**Figure 1:**
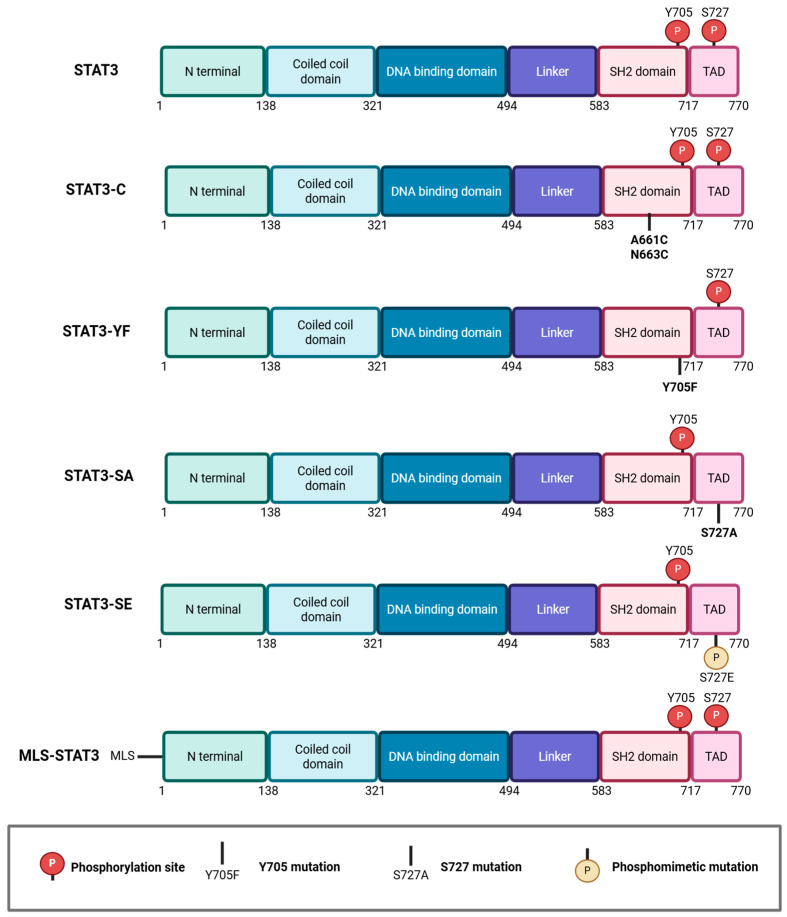
Schematic overview of STAT3 structural domains and mutant constructs, including mitochondrial-targeted STAT3, used to dissect the functional roles of S727 and Y705 phosphorylation, including STAT3-C (constitutively active STAT3), STAT3-YF (Y705F phosphorylation-defective mutant), STAT3-SA (S727A phosphorylation-defective mutant), STAT3-SE (S727E phosphomimetic mutant), and mitochondria-targeted STAT3 (MLS-STAT3, bearing a mitochondrial localization sequence). See text for details and references.

The functional distinction between Y705 and S727-dependent STAT3 activities has been dissected using phosphorylation defective mutants (Figure 1). Substitution of Y705 with phenylalanine (Y705F) abolishes STAT3 dimerization and transcriptional activity while preserving specific extranuclear functions [15,19,25,29,30]. In contrast, mutation of S727 to alanine (S727A) selectively disrupts non-canonical STAT3 activities at both mitochondria and the ER compartment, without affecting nuclear transcription [15,19,25,29,30,31]. Phospho-mimetic S727 mutants (S727D or S727E), as well as combinatorial mutants (Y705F/S727A or Y705F/S727E) were designed to isolate serine-driven outputs from canonical transcription [25,29,30]. In order to explore the tumor-transformation activities of constitutively active STAT3, two cysteine substitutions were introduced in the SH2 domain, promoting stable dimerization independently of Y705 phosphorylation, was generated. This STAT3C mutation results in persistent nuclear localization and transcriptional activity and is able to support tumor transformation of immortalized cells [3,12]. In vivo, STAT3C enhances breast cancer cell migration and metastases in mouse mammary tumor virus (MMTV)-Neu transgenic mice [3,32]. Fusion proteins carrying a mitochondrial localization sequence (MLS-STAT3), driving localization to the inner mitochondrial membrane, were additionally employed to explore mitochondrial functions [17,25] (Figure 1).

Breast cancer (BC) represents a clinically and biologically relevant context to apply this framework. STAT3 expression and activation are frequently elevated in mammary tumors and particularly in triple-negative breast cancer (TNBC), where constitutive STAT3 signaling and elevated STAT3 mRNA levels have been linked to aggressive behavior, metabolic plasticity, immune evasion, and therapeutic resistance [33,34,35,36]. Importantly, elevated STAT3 expression correlated with reduced relapse-free survival (RFS) both in BC in general and in TNBC patients [37]. However, no clear correlation was observed with the levels of pY705-STAT3 [38]. Interestingly, p-S727 STAT3 was increased in breast infiltrating ductal carcinoma and correlated with ER-negative status and tumor aggressiveness [39], while pY705-STAT3 expression was shown to correlate with longer overall survival (OS) and RFS in ER-positive breast tumors [40]. Moreover, STAT3 S727 and Y705 phosphorylation have been associated with distinct tumor cell phenotypes in TNBC patients, suggesting distinct functional roles in breast tumor biology [41]. In this vein, STAT3 activity in TNBC was reported to be predominantly driven by S727 phosphorylation with limited involvement of Y705 [42]. This pattern appears to be subtype-specific, as STAT3 expression and activation are rarely detected in HER2-positive and luminal breast tumors, underscoring a TNBC-specific reliance on p-S727 STAT3 signaling [42].

The reported findings support the idea that STAT3 S727 phosphorylation represents a functionally distinct and clinically relevant signaling axis that operates beyond canonical Y705-driven transcription and critically sustains tumor progression and adaptive responses, particularly in TNBC. We posit that the relative balance and subcellular distribution of p-S727 and p-Y705 STAT3 forms integrate context-dependent oncogenic inputs and therapeutic vulnerabilities. Accordingly, we propose that patient stratification models incorporating the p-S727/p-Y705 axis and the development of p-S727-specific inhibitors would be predicted to improve precision, efficacy and safety of STAT3-directed therapeutic interventions.

In support of this view, here we first summarize canonical Y705-mediated STAT3 signaling and its established roles in cancer (Section 2). We then discuss emerging non-canonical STAT3 functions, with particular emphasis on alternative subcellular localizations and the distinct biological activities associated with S727 phosphorylation (Section 3), focusing on cancer (Section 4), and then more specifically on BC and TNBC (Section 5). Finally, we review experimental evidence underlying the potential translational relevance of targeting p-S727 STAT3 signaling, particularly in mitochondria (Section 6) and in BC/TNBC (Section 7).

## 2. Canonical Y-705-Mediated STAT3 Signaling in Cancer

As mentioned above, in most instances constitutive STAT3 Y705 phosphorylation in cancer occurs downstream of aberrantly active upstream inducers, such as IL-6-type cytokines and growth factors. In this vein, particularly relevant is the original observation that STAT3 is required for tumor transformation downstream of the Src oncogene [43]. The oncogenic potential of canonical STAT3 signaling is largely attributable to its extensive transcriptional output, which mostly aligns with classical cancer hallmarks [8,10,44]. STAT3 promotes cell-cycle progression by inducing cyclins (e.g., cyclin D1/D2) and c-MYC, thereby facilitating the G1/S transition and sustaining proliferative signaling [1,45]. In parallel, STAT3 enhances survival and resistance to apoptosis by upregulating anti-apoptotic BCL-2 family members (BCL-2, BCL-XL, MCL-1) and inhibitors of apoptosis such as survivin (BIRC5), XIAP, and c-IAP2 [1,10]. Moreover, STAT3 participates in metabolic rewiring and interfaces with mitochondrial signaling pathways that shape apoptotic thresholds and transformation [10,11,46]. Both transcriptional and non-transcriptional outputs collectively blunt intrinsic and extrinsic apoptotic pathways, contributing to resistance to chemotherapy, radiotherapy, and targeted agents [8,10,11,12,24,44,47].

Canonical STAT3 signaling also plays a central role in tumor angiogenesis and metastatic dissemination. STAT3 directly induces Vascular Endothelial Growth Factor (VEGF) expression and, under hypoxic conditions, cooperates with Hypoxia-Inducible Factor 1α (HIF-1α) to promote neovascularization [8,10,45]. Of relevance, STAT3 constitutive activity was shown to induce a metabolic switch to aerobic glycolysis via the up-regulation of HIF-1α itself [46]. During invasion and metastasis, STAT3 interacts with epithelial–mesenchymal transition (EMT)-associated transcription factors and with TGF-β/SMAD signaling to repress epithelial markers such as E-cadherin and to induce mesenchymal markers, including twist, vimentin and N-cadherin. Simultaneously, STAT3 induces the expression of matrix metalloproteinases (MMP2, MMP9) [8,10,44,47], thus contributing to extra-cellular matrix remodeling, known to be crucial for tumor growth and metastases. These processes support local invasion, intravasation, and colonization at distant organs and are particularly relevant in aggressive breast cancer subtypes such as TNBC [44,47,48,49].

STAT3 signaling also regulates the maintenance of cancer stem cells (CSC), where its persistent activation sustains self-renewal, survival and tumor-initiating capacity [50]. Through the IL-6/JAK/STAT3 axis STAT3 induces the expression of stemness-associated genes, including OCT4, SOX2, NANOG, KLF4 and MYC thereby enhancing CSC self-renewal and plasticity, invasiveness and therapy resistance [50,51,52]. Finally, due to its significant correlation with the expression of CSC phenotype markers, STAT3 was proposed as a marker and regulator of CSCs, together with ΔNp63, particularly in the triple-negative subtype [52].

Beyond tumor cell–intrinsic functions, transcriptional STAT3 activities exert profound non-cell-autonomous effects within the tumor microenvironment. STAT3 activation in both immune (dendritic cells, macrophages, neutrophils, myeloid-derived suppressor cells) and mesenchymal components (fibroblasts, endothelial cells, mesenchymal stem cells) of the tumor stroma promotes an immunosuppressive and pro-tumorigenic milieu [1,8,44,45,48,53]. In cancer cells, STAT3 dampens antigen presentation, upregulates immune checkpoint molecules such as PD-L1, and drives secretion of IL-6, IL-10, and TGF-β, which in turn reinforce the expansion and activity of regulatory T cells and myeloid-derived suppressor cells [1,8,45,53]. This establishes a feed-forward loop in which tumor- and stroma-derived cytokines maintain chronic STAT3 activation, that, in turn, reciprocally stabilizes an immunosuppressive and inflammatory tumor-promoting microenvironment [1,8,45,53].

At the chromatin level, canonical Y705-dependent STAT3 dimers bind GAS motifs in promoters and distal regulatory elements. Genome-wide analyses show that STAT3-occupied regions frequently overlap with active enhancers enriched for p300/CBP, BRD4, and H3K27ac, indicating that STAT3 not only drives transcription initiation but also contributes to enhancer remodeling and higher-order chromatin organization [54]. Cooperative binding with NF-κB, AP-1, and nuclear hormone receptors further shapes STAT3-dependent transcriptional specificity, explaining how STAT3 can exert context-dependent oncogenic functions across diverse tumor types [10,54,55]. In breast cancer, including TNBC, these interactions integrate growth factor, hormone, and inflammatory cues into consolidated transcriptional programs that promote tumor growth, stemness, and therapeutic resistance [8,44,47].

Given its central role in tumor biology, STAT3 has long been pursued as a therapeutic target. Strategies include inhibiting upstream cytokines and their receptors (e.g., IL-6/IL-6R blockade), targeting JAK kinases, interfering with SH2-mediated STAT3 Y705 phosphorylation and dimerization, blocking DNA binding, or promoting STAT3 degradation [56,57,58]. Several small-molecule inhibitors have shown preclinical activity in TNBC and other malignancies, but clinical translation has been challenging due to issues of specificity, pharmacokinetics, and on-target toxicities [45,56,57,58]. Notably, most of these efforts have historically focused on Y705-mediated activities and canonical nuclear signaling, reflecting the generalized view that Y705 is the predominant driver of STAT3 oncogenic functions [4,10,56,57,58].

## 3. STAT3 Alternative Subcellular Localizations and Non-Canonical Functions

STAT3 phosphorylation on serine 727 (p-S727) has for decades been regarded as an accessory post-translational modification (PTM), primarily functioning to fine-tune signaling cascades initiated by tyrosine 705 (Y705) phosphorylation [43,59]. In this canonical framework, p-S727 was assigned a minor modulatory role in nuclear STAT3 signaling, where it was shown to enhance transcriptional efficiency by facilitating recruitment of transcriptional co-activators, stabilizing STAT3-containing transcriptional complexes, and promoting chromatin engagement at target gene loci [22]. Mechanistically, S727 phosphorylation was linked to increased interaction with histone acetyltransferases (HAT) such as p300/CBP, improved RNA Pol II recruitment, and enhanced transcriptional elongation, thereby maximizing the output of Y705-dependent STAT3 dimers without altering DNA-binding specificity or nuclear localization [22,23]. On the other hand, p-S727 STAT3 was also shown to antagonize subsequent Y705 phosphorylation, suggesting a potential negative-feedback mechanism. However, this initial observation was not further pursued [22,23,28].

This Y705-centric view was substantially broadened by evidence indicating that S727 phosphorylation can define qualitatively distinct non-canonical, non-nuclear STAT3 functions. A pivotal shift arose from studies reporting localization of STAT3 to mitochondria, where its p-S727 form was reported to regulate processes unrelated to transcription [17,21,60]. Seminal mutational studies and experimental overexpression indicated that mitochondrial STAT3 requires S727—but not Y705—phosphorylation to modulate electron transport chain (ETC) activity, particularly at complexes I and II, thereby enhancing oxidative phosphorylation while limiting excessive reactive oxygen species production [17,21,60]. At the molecular level, p-S727 STAT3 was shown to associate with mitochondrial membranes and interact with ETC components. These observations are consistent with its potential involvement in NADH oxidation, membrane potential, and ATP production [17,21,60]. These activities directly promote metabolic flexibility, resistance to oxidative stress, and oncogenic transformation downstream of *Ras* oncogenes and MAP kinases, particularly under conditions in which mitochondrial function becomes rate-limiting for tumor cell survival and proliferation [17,21,25,26,28]. Importantly, these effects occur independently of STAT3 DNA binding or transcriptional activity, challenging the paradigm of STAT3 as an exclusively nuclear transcription factor [17,21,60].

Although early models proposed that S727 phosphorylation is required for mitochondrial recruitment of STAT3 [60], subsequent experimental evidence has challenged this view. Studies in primary mouse tissues demonstrated that loss of S727 phosphorylation did not abolish STAT3 mitochondrial localization [61]. Similar observations were reported in proliferating embryonic zebrafish stem cell niches and mouse stem cells, where Y705—but not S727—appeared to influence sub-mitochondrial localization, but S727 alone was required for transcriptional activation of mitochondrial genes and proliferation [62]. Contrasting results describing reduced mitochondrial STAT3 levels in S727A mutants suggest that perhaps the contribution of S727 phosphorylation may be context-dependent and influenced by metabolic state or chaperone availability, including GRIM-19 [60]. Marié and colleagues reported that STAT3 mitochondrial targeting is governed by both the amino-terminal domain (aa 1–132), well known to engage in protein–protein interactions [16], and the carboxyl-terminal α-helical region (aa 536–583) of the linker domain [61]. The former is required for effective mitochondrial localization but not sufficient to confer targeting on its own. In contrast, the latter contains an internal mitochondrial targeting sequence that is sufficient for translocation. However, the authors did not test the relevance of Y705 phosphorylation in this context.

Beyond the modulation of mitochondrial respiration and the transcriptional activation of mitochondrial genes [21,63], p-S727 STAT3 has been implicated in broader metabolic rewiring. Serine phosphorylation integrates signals from MAPK, mTOR, CDK, and stress-activated kinases, positioning STAT3 as a downstream effector of growth factor signaling, nutrient availability, and cellular stress responses [21,22,28,60,64]. Through these pathways, evidence suggests that p-S727 STAT3 contributes to the coordination of glycolysis, glutamine metabolism, and mitochondrial biogenesis, thereby aligning metabolic programs with proliferative and survival pathways, both in physiological and pathological contexts [17,21,60]. Recent transcriptomic and functional studies further support the notion that p-S727 drives distinct, Y705-independent gene-expression programs associated not only with tumor aggressiveness but also with metabolic adaptation [65,66].

It is worth mentioning that some studies have questioned the extent, regulation, and relevance of mitochondrial STAT3 localization, highlighting technical limitations of mitochondrial fractionation leading to contamination by cytosolic or ER-associated membranes, potentially leading to overestimation of mitochondrial STAT3 pools [20,67]. Rigorous validation of fraction purity using multiple organelle-specific markers and complementary approaches, such as high-resolution imaging, is therefore essential. In addition, variability in antibody specificity for p-S727 detection represents a significant concern. Phospho-specific antibodies may differ in sensitivity, epitope recognition under denaturing versus native conditions, and cross-reactivity with unrelated phospho-proteins. Inadequate validation—such as failure to use S727A phospho-deficient mutants, phosphatase treatment controls, or orthogonal detection methods—can complicate interpretation of subcellular localization and functional studies. Together, these technical considerations underscore the need for standardized validation strategies and complementary methodologies when investigating p-S727 STAT3, particularly in the context of extranuclear functions. Indeed, demonstration of the functional mechanisms explaining intramitochondrial STAT3 functions remains experimentally challenging, particularly given the low abundance of STAT3 relative to core mitochondrial proteins [20,67]. One potential source of artifacts is that early studies mostly relied on the over-expression of wild-type and mutant STAT3 proteins artificially tagged to the mitochondrion thanks to a fusion with a mitochondrial signaling peptide at the N-terminus [17,68,69,70]. Recently, via rigorous subcellular fractionation and super-resolution imaging analyses, Su and colleagues have shown that endogenous STAT3 is lowly represented in purified mitochondrial fractions while abundantly localized to the MAMs [20]. These findings raise the possibility that at least part of the phenotypes attributed to mitochondrial STAT3 may originate from functions exerted at the ER-mitochondria contact sites rather than within the mitochondrial matrix.

**Figure 2:**
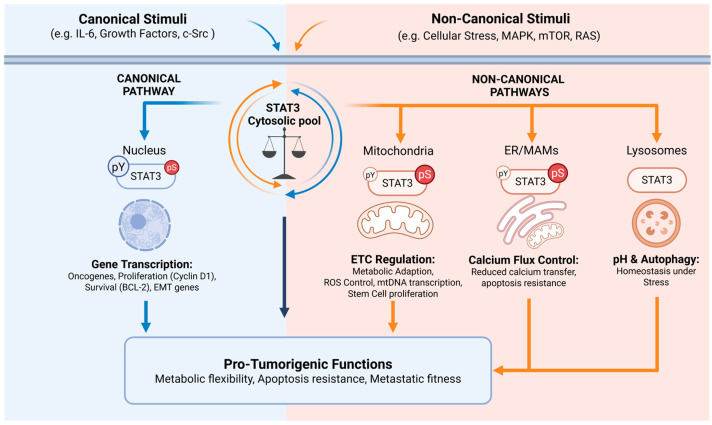
**Comprehensive model depicting the balance between canonical and non-canonical STAT3 functions. Left (Blue):** Canonical activation by stimuli such as IL-6, growth factors, or c-Src leads to tyrosine phosphorylation (p-Y705) and nuclear translocation. **Right (Orange):** Non-canonical activation triggered by cellular stress and kinase signaling (MAPK, mTOR, RAS) directs STAT3 to various organelles. In the mitochondria, STAT3 regulates the electron transport chain (ETC), ROS production, mtDNA transcription, and SC proliferation; at the ER/MAMs, it modulates calcium flux and apoptosis resistance; finally, in lysosomes it maintains lysosomal pH, while in the cytoplasm it inhibits autophagy.

Consistently, our group has identified STAT3 as an ER- and MAM-localized protein regulating calcium fluxes between the ER and mitochondria through interaction with the IP3R3 calcium channel and its degradation [19], thereby controlling mitochondrial Ca^2+^ uptake, permeability transition pore opening, and apoptotic sensitivity [19,71,72]. IP3R3 degradation and apoptosis protection, but not ER/MAM localization or interaction with IP3R3, require S727 phosphorylation [19]. These findings support an integrative model where STAT3 acts as a versatile signaling hub whose non-nuclear activities extend beyond mitochondria to include ER and MAM domains [18,19,20] (Figure 2).

Alongside its mitochondrial, ER and MAM functions, STAT3 was shown to localize to distinct cytoplasmic locations. Indeed, cytoplasmic STAT3 has been shown to repress autophagy by interacting with PKR via its SH2 domain and inhibiting PKR-mediated eIF2α phosphorylation [73]. Y705 phosphorylation was not required for this activity, while S727 phosphorylation was not assessed. More recently, lysosome-focused studies revealed that non phosphorylated STAT3 dynamically associates to the cytosolic surface of LAMP1/LAMP2-positive lysosomes, where it binds the V-ATPase complex via its coiled-coil domain [74]. This positioning was proposed to enable STAT3 to participate in the regulation of proton gradients by stimulating V-ATPase activity, thereby maintaining an alkaline cytosolic pH and an acidic lysosomal lumen, ultimately promoting tumor cell homeostasis under metabolic stress. Pharmacological perturbations of lysosomes further support this view: cationic amphiphilic drugs that dissipate lysosomal acidity trigger cytosolic acidification, dephosphorylation of STAT3, and its redistribution toward lysosomes, thereby functionally inactivating STAT3 oncogenic signaling [75].

Together, these findings extend the spatial map of non-canonical STAT3 beyond mitochondria and ER/MAMs to include a lysosome-related regulatory axis through which STAT3 integrates pH homeostasis, autophagy, and stress signaling within the cytoplasm. It is worth noting that also the canonical transcriptional activity of STAT3 was shown to take part in lysosome regulation. Indeed, STAT3 induces the expression of cathepsins, achieving either cell adaptation to protein overload [76] or lysosome-mediated programmed cell death in the context of post-lactational involution of the mammary gland [76,77,78]. The compartment-specific functions of p-Y705 and p-S727 STAT3 are summarized in Table 1. Although distinct roles of these PTMs are evident in certain cellular compartments (nucleus, mitochondria, and ER-MAMs), further investigation is needed to clarify whether STAT3 alone is functionally sufficient or whether specific PTMs are required to confer differential, compartment-dependent activities where they have not been directly investigated.

Further complicating the picture, several studies suggest that nuclear, cytosolic, ER-associated, and mitochondrial pools of STAT3, whether phosphorylated on S727 or not, can coexist and are subject to dynamic and sometimes reciprocal regulation [69,75,79]. In this frame, p-S727 acts as a molecular integrator of environmental cues rather than a fixed determinant of subcellular localization and function. It is moreover worth noting that at present the actual localization of S727 phosphorylation events has not been investigated. Determining whether phosphorylation is also localized at the specific compartments where p-S727 is physically detected and functionally characterized, or whether there is a cytoplasmic pool of p-S727 STAT3 that is then sorted to the different cellular compartments in response to specific cues will be instrumental to understand how its different functions are regulated and how they interact with each other.

Collectively, these findings underline an ongoing debate regarding the mechanistic scope of p-S727 STAT3 in cancer biology. Rather than representing a binary nuclear-versus-mitochondrial switch, S727 phosphorylation is increasingly recognized as a dynamic regulatory layer that modulates STAT3 functions across multiple cellular compartments, metabolic requirements and signaling axes, often synergizing with, but sometimes opposing, the canonical functions of nuclear STAT3.

**Table 1:** **Compartment-specific functions of p-Y705 and p-S727 STAT3.** Summarized compartment-specific functions of p-Y705 and p-S727 STAT3. p-Y705 primarily drives dimerization, nuclear localization, and transcriptional activation, whereas p-S727 enhances transcriptional efficiency and mediates transcription-independent functions, including regulation of oxidative metabolism, calcium signaling, and stress responses. In contrast, the role of p-S727 in cytoplasmic inhibition of autophagy was not investigated, while it was ruled out in the stimulation of lysosomal V-ATPase activity. ETC, electron transport chain; ROS, reactive oxygen species; ER, endoplasmic reticulum; MAMs, mitochondria-associated membranes.

Cellular Compartment	p-Y705	p-S727	Reference(s)
Nucleus	STAT3 dimerization, nuclear localization and transcriptional activation	Enhances transcriptional output via co-activator recruitment and transcriptional elongation; possible negative feedback on Y705	[1,2,3,4,8,22,23,28,43,59]
Mitochondria	Not required for mitochondrial metabolic activity; possible involvement in sub-mitochondrial localization	Regulates ETC activity and oxidative phosphorylation; limits ROS; transcription-independent metabolic control	[17,21,25,60,61,62]
ER-MAMs	No defined direct role	Controls ER–mitochondrial Ca^2+^ flux and apoptotic sensitivity via IP3R3 regulation	[18,19,20]
Cytosol (autophagy repression)	Not involved	Not investigated	[74,75,76,77,78]
Lysosomes	Not involved	Not involved	[74,75,76,77,78]

## 4. Roles of STAT3 S727 Phosphorylation in Cancer

In recent years, STAT3 serine 727 phosphorylation has emerged as a clinically relevant signaling event in cancer, with multiple studies associating it with tumor grade, patient outcome, and metastatic progression. The main clinical findings are discussed below and summarized in Table 2.

The first evidence that STAT3 S727 phosphorylation can actively promote tumorigenesis independently of its Y705 counterpart emerged from the work of Qin and colleagues demonstrating a pro-oncogenic role of a phosphomimetic S727E mutant [30]. In their work, this mutation in combination with a Y705F mutation that abolishes Y phosphorylation conferred significant in vitro and in vivo growth advantage to human prostate cancer (PCa) LNCaP cells, potentially mediated by downstream activation of c-MYC, MCL-1, and survivin. Moreover, the Y705F/S727E mutant—but not a Y705F/S727A mutant where also S727 phosphorylation is abolished—induced anchorage-independent growth in non-tumorigenic prostate epithelial RWPE-1 cells, further supporting a pro-tumorigenic role for STAT3 S727 phosphorylation [30]. Accordingly, a positive correlation between the levels of p-S727 STAT3 and Gleason score was observed in prostate cancer tissues [30]. The finding that S727, but not Y705, phosphorylation is required for *Ras*-mediated tumor transformation reported by Gough et al. [21] is in line with these observations.

More recently, high levels of cytoplasmic p-S727 STAT3 in the PCa stroma were identified as an independent prognostic marker associated with reduced OS and 5-year metastasis-free survival (MFS) following androgen deprivation therapy (ADT) in a cohort of 111 patients with advanced PCa [80]. Consistently, two independent studies reported that elevated p-S727 STAT3 was associated with higher Gleason scores [81,82]. Moreover, Thaper et al. demonstrated that STAT3 inhibition enhanced the sensitivity of PCa LNCaP cells to the androgen receptor (AR) inhibitor enzalutamide (ENZ) and that cell lines derived from ENZ-resistant tumors displayed up-regulation of p-S727, but not of pY705-STAT3, correlating with enhanced interactions with the AR and consequent increase in its activity [83]. Altogether, these findings suggest a role for p-S727 STAT3 in acquired therapeutic resistance and metastatic progression of PCa.

STAT3 S727 phosphorylation has also been linked to disease progression in gynecological malignancies. Indeed, in cervical intraepithelial neoplasia (CIN), p-S727 STAT3 levels correlated with lesion grade and expression of the proliferation marker Ki-67 in 56 untreated patients [84]. Similarly, p-S727 STAT3 was highly expressed in seven advanced-stage human endometrial tumor samples, whereas Y705 phosphorylation was undetectable [85]. Moreover, several human endometrial cancer (EC) cell lines exhibited strong cytosolic p-S727 STAT3 expression in the absence of Y705 phosphorylation, suggesting a distinctive role for S727 phosphorylation in EC development [85].

A comparable phosphorylation pattern has been reported in melanoma. STAT3 S727 was found to be constitutively phosphorylated in seven out of seven human melanoma cell lines analyzed, downstream of the bRaf–MEK–ERK1/2 pathway, while p-Y705 STAT3 levels were much more variable [86]. Moreover, p-S727—but not p-Y705—STAT3 was present in five out of nine in situ lesions of acral lentiginous melanoma (ALM) patients, whereas both phosphorylation events were detected in dermally invasive ALM lesions [86].

A Y705-independent prognostic value for STAT3 S727 phosphorylation was also demonstrated in clear cell renal cell carcinoma (ccRCC). In a cohort of 98 patients with 100-month follow-up, high p-S727 STAT3 levels correlated with higher Fuhrman grade and poorer OS [87]. Consistently, in an independent cohort of 82 ccRCC patients followed for 10 years after nephrectomy, elevated p-S727 STAT3 levels—both nuclear (*p* = 0.002) and cytosolic (*p* = 0.040)—were associated with reduced OS [88].

In lung cancer (LC), immunohistochemical (IHC) analysis of 40 primary tumor specimens revealed that high p-S727 STAT3 levels, together with elevated leukemia inhibitory factor receptor (LIFR) expression, significantly correlated with higher tumor grade and reduced OS [89]. In the same study, STAT3 S727 and Y705 phosphorylation were shown to differentially regulate EMT and mesenchymal–epithelial transition (MET) programs, with p-Y705 STAT3 enriched in primary tumors and p-S727 STAT3 predominant in metastatic regions [89]. In this context, the LIFR/p-ERK/p-S727-STAT3 signaling axis drove MET, whereas EMT was associated with IL6R/p-Y705 STAT3 signaling in bone marrow-derived mesenchymal stem cells (BM-MSCs). Importantly, these effects were validated in vivo using a bone homing assay with human lung adenocarcinoma A549 cells, and abolished upon STAT3 inactivation [89].

Finally, in glioblastoma (GBM), STAT3 S727 phosphorylation was detected in 100% and 70% of tumors from a cohort of 30 and 88 patients, respectively [90,91], being associated with poor OS in both univariate and multivariate analyses [91]. Patients exhibiting high levels of both p-S727 and p-Y705 STAT3 experienced the worst clinical outcomes, suggesting potential synergistic effects [91]. Interestingly, in temozolomide (TMZ)-resistant U373 and U251 glioma cells, p-S727 STAT3 levels were increased, whereas p-Y705 STAT3 levels were reduced. STAT3 silencing restored TMZ sensitivity, supporting p-S727 STAT3 as a promising therapeutic target in TMZ-resistant gliomas [92]. Consistently, STAT3 S727 phosphorylation correlated with intrinsic radioresistance across 15 GBM cell lines, and treatment with the multi-kinase inhibitor Gö6976 restored radiosensitivity at least in part through selective down-modulation of p-S727 STAT3 in Y705-negative or weakly positive GBM cells, highlighting S727 phosphorylation as a potential therapeutic target in this context [90].

In conclusion, while most of the clinical studies were correlative in nature, their convergence with functional genetic and pharmacological data supports a casual contribution of p-S727 STAT3 to aggressive tumor phenotypes.

**Table 2:** List of tumor types where p-S727 STAT3 levels were analyzed and correlated with tumor grade, overall survival (OS), or metastasis-free survival (MFS). PCa, prostate cancer; CIN, cervical intraepithelial neoplasia; ALM, acral lentiginous melanoma; ccRCC, clear cell renal cell carcinoma; LC, lung cancer, GBM, glioblastoma; BC, breast cancer; TNBC, triple negative breast cancer; POS, positive; NEG, negative; N/A, not available.

Tumor Type	Patients (n)	p-S727 STAT3+	Correlation with Grade	Correlation with OS	Correlation with MFS	Reference
PCa	20	65%	Pos (*p* = 0.05)	N/A	N/A	[30]
PCa	111	49%	N/A	Neg (*p* = 0.013)	Neg (*p* = 0.034)	[80]
CIN	56	100%	Pos (*p* < 0.001)	N/A	N/A	[84]
ALM	15	73%	N/A	N/A	N/A	[86]
ccRCC	98	N/A	Pos (*p* < 0.01)	Neg (*p* < 0.001)	N/A	[87]
ccRCC	82	N/A	N/A	Neg (*p* = 0.002)	N/A	[88]
LC	40	N/A	Pos	Neg (*p* = 0.017)	N/A	[89]
GBM	30	100%	N/A	Not affected	N/A	[90]
GBM	88	70%	N/A	Neg (*p* = 0.002)	N/A	[91]
BC	68	62%	Pos (*p* = 0.024)	N/A	N/A	[39]
BC	48	56%	N/A	N/A	N/A	[26]
TNBC	173	N/A	Neg (*p* = 0.016)	Not affected	N/A	[41]
TNBC	76	80%	N/A	N/A	N/A	[42]

## 5. p-S727 STAT3 Activities in BC and TNBC

Although as described above p-S727 is likely to exert pro-tumoral action in multiple malignancies, it appears to be particularly enriched and functionally relevant in TNBC, which we therefore dissect in better detail in this section.

The clinical relevance of STAT3 S727 phosphorylation in BC remained largely unexplored until Yeh and colleagues reported that high nuclear and cytoplasmic p-S727 STAT3 levels—detected in 62% of infiltrating ductal carcinoma samples compared with adjacent non-tumoral tissues—significantly correlated with estrogen receptor (ER)-negative status, larger tumor size, and advanced disease stage [39]. This inverse correlation was further validated in vitro using a panel of BC cell lines, and substantiated by the observation that knocking down ERα in ER-positive MCF7 cells increased p-S727 STAT3 levels [39].

In contrast, a positive correlation between nuclear p-S727 STAT3 and progesterone receptor (PR) expression was reported in a cohort of 39 infiltrating ductal breast carcinomas (*p* = 0.027) [26]. Mechanistically, the synthetic progestin medroxyprogesterone acetate (MPA) induced STAT3 S727 phosphorylation by activating the p42/p44 MAPK signaling pathway and promoted its nuclear localization. This event was required for STAT3 binding to the *cyclin D1* promoter in vivo, thereby stimulating BC cell proliferation [26]. Notably, MPA-induced growth was abrogated by expression of the STAT3 S727A mutant in both murine progestin-dependent C4HD cells and human luminal A T-47D BC cells, resulting in G1 cell-cycle arrest [26].

A recent study analyzing 173 TNBC patient samples demonstrated that STAT3 S727 and Y705 phosphorylation occur independently in 34% of cases, implying distinct functional roles in breast tumor biology [41]. The aggressive basal-like tumors were characterized by high p-S727 and low p-Y705 STAT3 levels, whereas dual ERβ and AR positivity correlated with low p-S727 and high p-Y705 STAT3 expression [41]. Interestingly, both STAT3 phosphorylation events were associated with lower tumor size and clinical stage but not with improved or reduced survival, suggesting that high STAT3 S727/Y705 phosphorylation can skew the good prognosis conferred by clinicopathological characteristics [41], possibly due to the ability of STAT3 to induce drug resistance [93].

STAT3 S727 phosphorylation has recently been linked to STAT3 activation and dimerization in a Y705-independent manner, as demonstrated using a bioluminescence resonance energy transfer (BRET) biosensor in luminal A MCF7 and TNBC MDA-MB-231 cells [42], consistent with previous observations in chronic lymphocytic leukemia (CLL) [94]. Accordingly, MCF7 cells expressing the STAT3 S727A mutant displayed significantly reduced proliferation and clonogenic capacity compared with cells expressing the Y705F mutant [42]. In line with these findings, a retrospective IHC analysis of 76 TNBC patient samples revealed that over 92% of STAT3-positive tumors exhibited constitutive S727 phosphorylation, whereas Y705 phosphorylation was detected in only 15% of cases [42]. In contrast, STAT3 expression—and phosphorylation—was absent or weakly cytoplasmic in HER2-positive and luminal BC subtypes, supporting a subtype-specific role of STAT3 and its S727 phosphorylated form in TNBC [42].

Mitochondria-localized STAT3 S727 phosphorylation was shown to promote TNBC growth and metastasis in vivo in a Y705-independent manner using the murine 4T1 model [25]. 4T1 cells expressing a mitochondrially tagged MLS-STAT3 S727A mutant formed significantly fewer colonies in soft agar, whereas expression of the STAT3 S727D phosphomimetic mutant markedly increased anchorage-independent growth. In vivo, tumors derived from cells expressing MLS-STAT3 S727A/Y705F were smaller and exhibited reduced liver and lung metastasis compared with those expressing MLS-STAT3 S727D/Y705F. Importantly, inactivation of STAT3 transcriptional functions through Y705F mutation, SH2 disruption, or deletion of the DNA-binding domain did not affect tumor growth, supporting a transcription-independent role for mitochondrial STAT3 in TNBC [25]. Accordingly, a construct lacking the nuclear localization sequence enhanced mitochondrial STAT3–driven invasion in Matrigel assays, confirming that nuclear activities are not involved and suggesting reciprocal regulation between the nuclear and mitochondrial forms. Mechanistically, p-S727 STAT3 improved mitochondrial complex I coupling, thereby reducing ROS production. These effects were observed under hypoxic but not normoxic conditions, supporting a model in which mitochondrial phosphorylated STAT3 S727 functions as a rheostat to fine-tune ROS levels to promote TNBC cell survival and metastatic fitness rather than apoptosis [25]. Despite not experimentally proven, the observation that mitochondrial p-S727 STAT3 is required for the proliferation of stem cells [62] might extend this feature to de-differentiated cancer cells.

STAT3 S727 phosphorylation has also emerged as a critical determinant for life/death decisions in BC cells upon silencing of the lactate dehydrogenase C (LDHC) gene [95], which in turn was previously associated with poor OS in basal-like and HER2-enriched breast tumors [96]. Indeed, LDHC knockdown markedly impaired the survival of basal-like MDA-MB-468 and BT-549 TNBC cells through p-S727 STAT3 downregulation, inducing DNA damage accumulation, microtubule destabilization, and cell-cycle dysregulation through p-S727 STAT3 downregulation. In contrast, LDHC silencing in HER2-enriched HCC-1954 cells increased p-S727 STAT3 levels and conferred resistance to cell death, which was reversed by STAT3 silencing [95], highlighting how regulatory networks are determined by biological conditions. p-S727 STAT3 was also shown to form a transcriptional complex with BCL9 at enhancer regions of target genes, driving invasive progression in HER2-enriched SUM225 and DCIS.COM BC cell lines [97].

Furthermore, STAT3 phosphorylation downstream of the AKT-mTOR signaling pathway was implicated in the activation of the CC motif chemokine receptor 1 (CCR1) promoter following epidermal EGF stimulation, thereby promoting invasion and metastasis in MDA-MB-231 TNBC cells [98]. In turn, CCR1 silencing reduced invasive sprouting in 3D spheroid cultures in vitro and diminished MDA-MB-231 lung metastatic colonization in vivo [98].

Finally, STAT3 S727 phosphorylation was found to be upregulated by approximately 1.8-fold in the side population of luminal A MCF7 cells—a rare subpopulation enriched in cancer stem-like cells with enhanced clonogenic capacity in vitro and increased tumorigenicity in vivo—further supporting a role for p-S727 STAT3 in breast cancer aggressiveness [27].

## 6. Targeting Mitochondria-Associated STAT3 S727 Signaling

Substantial efforts have been devoted to pharmacologically target mitochondrial STAT3, leading to the development of multiple small molecules and experimental strategies aimed at disrupting its non-canonical functions.

The first of such agents was phospho-valproic acid (P-V; MDC-1112), which inhibited the growth of human pancreatic cancer xenografts both by inhibiting STAT3 Y705 phosphorylation by JAK and Src kinases and by reducing total and S727-phosphorylated STAT3 levels in mitochondria, leading to increased mitochondrial ROS accumulation and induction of apoptosis. [99]. Reduced STAT3 in mitochondria could be due to P-V-mediated impairment of STAT3 interactions with HSP90 and TOM20, the latter involved in STAT3 mitochondrial translocation in addition to GRIM-19 [100]. Importantly, inhibition of mitochondrial STAT3 was required for P-V antitumor activity, as overexpression of mitochondrially targeted STAT3 rescued tumor growth in vivo. Although P-V reduced IL-6–induced STAT3 Y705 phosphorylation in BxPC-3 cells, it failed to suppress the growth of tumors overexpressing either wild-type or Y705F STAT3, indirectly suggesting that its antitumor efficacy might depend on targeting mitochondrial STAT3 S727 phosphorylation rather than canonical Y705-mediated signaling [99].

Another strategy to target mitochondria-localized STAT3 involved conjugating the mitochondria-targeting fluorophore *N,N*-diethyl-7-aminocoumarin to a benzo[b]thiophene 1,1-dioxide (BTP) moiety, generating compound 7a [101]. This compound co-localized with the mitochondrial marker MitoTracker in MDA-MB-231 TNBC and luminal A MCF7 cells, although partial lysosomal localization was also observed. Notably, compound 7a dose-dependently inhibited both STAT3 S727 and Y705 phosphorylation in MDA-MB-231 cells without affecting upstream STAT3 regulators such as p-JAK2, p-Src, or p-ERK1/2 [101]. 7a suppressed in vitro MDA-MB-231 cell viability, mediated by downregulation of STAT3 target genes such as BCL-2 and cyclin D1, increased ROS production and activated the mitochondrial apoptotic pathway. Antitumor activity and inhibition of p-Y705 STAT3 were further confirmed in syngeneic 4T1 tumors in vivo [101].

Mitochondrial STAT3 has also been implicated in the toxicity and antitumor activity of the STAT3 inhibitor OPB-51602 [102], which was previously shown to inhibit the growth of DU145 PCa cancer cells by inducing mitochondrial dysfunction and proteotoxic aggregates formation through SH2-domain binding and dual inhibition of STAT3 phosphorylation on both S727 and Y705 [103]. The related compound OPB-31121 displayed 100–1000-fold higher potency than other STAT3 inhibitors (i.e., Cryptotanshinone and S3I.201) in suppressing S727 and Y705 phosphorylation and proliferation of DU145 and LNCaP PCa cells, likely due to its ability to occupy a broader and distinct hydrophobic region of the STAT3 SH2 domain [104]. However, OPB-31121 failed in a phase 1 clinical trial in subjects with advanced solid tumors due to adverse effects and therapeutic resistance [58,105]. Brambilla and colleagues later demonstrated that OPB-51602 inhibits mitochondrial complex I, leading to ROS accumulation, mitophagy, cytoskeletal remodeling, and cell death in A549 non-small cell lung carcinoma (NSCLC) and in MDA-MB-468 and -231 TNBC cells [102]. However, partial residual sensitivity to the compound in STAT3 knockout cells and technical limitations in isolating pure mitochondrial fractions suggest that OPB-51602 effects may not be exclusively mediated by mitochondrial STAT3.

More recently, a potentially more selective strategy to target STAT3 S727 phosphorylation was proposed by Wan and colleagues using Sculponeatin A (sptA) [106]. STAT3 was identified among the top 10 predicted targets of this compound, which indeed selectively reduced S727—but not Y705—phosphorylation in whole-cell lysates and purified mitochondrial fractions of H460, A549 and PC9 NSCLC cells, also confirmed by immunofluorescence analysis [106]. SptA preferentially inhibited proliferation of NSCLC cells over normal lung epithelial cells and reduced tumor growth in zebrafish and mouse A549 xenograft models, accompanied by decreased p-S727 and total STAT3 levels [106]. Mechanistically, sptA promoted proteasomal degradation of mitochondrial STAT3 via WWP2-mediated ubiquitination, a process supported by molecular dynamics simulations showing enhanced STAT3–WWP2 binding. Overexpression of mitochondrially targeted STAT3 antagonized sptA-induced mitochondrial dysfunction and rescued cell viability, confirming mitochondrial STAT3 as a critical target of sptA. Although sptA showed no overt hepatotoxicity, cardiotoxic effects were observed at higher doses in zebrafish models [106]. Notably, sptA was also reported to induce ferroptosis in breast cancer cells, suggesting broader therapeutic potential beyond NSCLC [107].

Despite the paucity of specific inhibitors, these studies confirm an important role of mitochondrial pS727 STAT3 in driving cancer, supporting efforts to develop specific, effective inhibitors.

## 7. Targeting STAT3 S727 Phosphorylation in TNBC

Several STAT3 inhibitors have shown efficacy in TNBC models in vitro and in vivo [33,56,58]. In this section, we specifically focus on therapeutic strategies that modulate STAT3 S727 phosphorylation in TNBC, with the main results summarized in Table 3. Of note, although compound 7a and OPB-51602 were discussed above in the context of mitochondrial p-S727 STAT3 targeting, both agents were also evaluated in TNBC models and are therefore included in Table 3 as compounds interfering with STAT3 S727 signaling in TNBC [101,102]. While the present section focuses on the effects of S727 inhibition in TNBC, for a broader overview of STAT3 inhibitors—mainly directed against Y705 phosphorylation—we refer readers to the recent comprehensive review by Berkley and colleagues [58].

The rationale of targeting STAT3 S727 phosphorylation in TNBC was initially established by Dimri and colleagues [42], who demonstrated that the anti-helminth drug compound Niclosamide (NSA) significantly reduced primary tumor growth and distant metastasis in an orthotopic xenograft model of MDA-MB-231 TNBC cells. Mechanistically, NSA was shown to prevent STAT3 dimer formation in a S727-dependent way in live cells while displaying inhibitory activity on both STAT3 phosphorylation events. In contrast, the widely used STAT3 inhibitor Stattic, an SH2-binding compound that inhibits both Y705 phosphorylation and dimerization, preferentially inhibited Y705 phosphorylation and failed to achieve comparable effects [42]. However, niclosamide is known to target multiple signaling pathways—including Wnt/β-catenin, mTORC1, NF-κB, and Notch—which raises the possibility of off-target effects contributing to STAT3 dimer disruption and tumor suppression and disqualify it as a clinically suitable compound [108].

The benzothiazole-based compound B19 has been reported to simultaneously inhibit STAT3 S727 and Y705 phosphorylation, thereby suppressing STAT3-dependent transcription of c-MYC and MCL-1 and inducing G2/M cell-cycle arrest and apoptosis in MDA-MB-468 TNBC and HEL erythroleukemia cells [109]. Molecular docking analyses, along with surface plasmon resonance (SPR) and fluorescence polarization (FP) assays, revealed that like Stattic B19 binds the STAT3 SH2 domain, at residues Leu706–Phe710. It is moreover poorly soluble, and its oral viability would require considerable optimization to improve pharmacokinetic properties and in vivo stability [109].

A synthetic derivative of the SH2-domain STAT3 inhibitor SLSI-1, named SLSI-1216, was shown to dose-dependently inhibit both STAT3 S727 and Y705 phosphorylation in MDA-MB-231 TNBC cells [37]. SLSI-1216 effectively suppressed the in vitro growth of MDA-MB-231 and HCC38 TNBC cells, while displaying an approximately 10-fold higher IC_50_ in non-tumorigenic human mammary epithelial MCF-10A cells [37]. Moreover, SLSI-1216 exhibited antitumor efficacy comparable to paclitaxel in an MDA-MB-231 xenograft model, where IHC analysis confirmed p-Y705 STAT3 downregulation in treated tumors; STAT3 S727 phosphorylation was not assessed in this setting [37].

SMY002, a naphthalene-based compound derived from selective estrogen receptor modulator (SERM) scaffolds, demonstrated potent inhibition of primary tumor growth and lung metastasis formation in a syngeneic 4T1 TNBC mouse model by suppressing STAT3 phosphorylation, dimerization, nuclear localization, and transcriptional activity [110]. Notably, SMY002 exhibited selective cytotoxicity toward mouse 4T1 and human MDA-MB-468 and -231 TNBC cells, while sparing normal human mammary epithelial MCF-10A cells [110]. However, it remains unclear whether SMY002-mediated inhibition of STAT3 phosphorylation was specifically assessed at Y705, as the antibody described in this paper targets p-S727 rather than p-Y705, raising some doubts about the author’s conclusions that antitumor activities are due to inhibition of Y705-dependent STAT3 activation.

A SMY002 analog with improved STAT3 SH2-domain affinity, RDp002, was subsequently developed and shown to inhibit TNBC cell proliferation, survival, invasion, and migration by inducing G0/G1 cell-cycle arrest and lysosome-dependent cell death through dual suppression of STAT3 S727 and Y705 phosphorylation [111]. RDp002 exhibited minimal cytotoxicity in normal mammary epithelial MCF-10A cells and human kidney epithelial HEK-293T cells, while also demonstrating activity in multiple myeloma (MM) cell lines, including U266 and MM.1S [111]. Importantly, RDp002 induced significant tumor regression in MDA-MB-468 xenograft models, with effective inhibition of both STAT3 phosphorylation events confirmed by IHC and Western blot analysis [111]. This compound also impaired lung dissemination of the TNBC 4T1 cells in a syngeneic mouse model.

More recently, Chen and colleagues developed YY002, a highly selective SH2-domain STAT3 inhibitor capable of simultaneously suppressing STAT3 S727 and Y705 phosphorylation at nanomolar concentrations without affecting the activity of known upstream STAT3 kinases, including ERK1, JNK1, SRC, p38δ, EGFR, JAK1, and JAK2 [112]. Mechanistically, YY002 inhibited both nuclear p-Y705 STAT3 signaling and mitochondrial p-S727 STAT3 functions, resulting in pronounced cell death in MDA-MB-231 and MDA-MB-468 TNBC cells. YY002 also exhibited superior antitumor efficacy compared with the orally available STAT3 inhibitor napabucasin (BBI608) in an MDA-MB-231 xenograft model. The latter was in turn previously shown to attenuate drug resistance of the ER+ MCF7 cells via down-regulating p-S727 and p-Y705 STAT3 levels [112,113]. Similar antitumor and anti-metastatic effects were observed in an orthotopic pancreatic cancer xenograft model, where YY002 reduced tumor growth and metastasis in a dose-dependent manner and significantly prolonged survival [112]. Moreover, two novel orally bioavailable dual STAT3 phosphorylation inhibitors directed against the SH2-domain were recently proven to be effective in preventing tumor growth of gastric and pancreatic cancer xenograft models through the inhibition of both STAT3 nuclear- and mitochondrial-related pro-tumorigenic functions [114,115].

Finally, Zheng and colleagues demonstrated that dual inhibition of STAT3 S727 and Y705 phosphorylation using the saponin pulchinenoside E2 (PSE2) in MDA-MB-231 and HS-578T TNBC cells significantly suppressed lung and liver metastases in a xenograft model [116]. PSE2 displayed greater anti-metastatic efficacy than Stattic, as evidenced by a reduced number of metastatic nodules and more potent inhibition of both p-S727 and p-Y705 STAT3 in metastatic lesions, as assessed by IHC [116]. Importantly, the anti-metastatic effects of PSE2 were restricted to the TNBC subtype, as no significant activity was observed in luminal A MCF-7 and T47D BC cells or in normal MCF-10A cells, whereas migration and invasion were markedly suppressed in multiple TNBC cell lines, including MDA-MB-231 and -468, HS-578T, and BT-549 [116].

Overall, the data reviewed identify STAT3 S727 phosphorylation as a functionally distinct and therapeutically relevant vulnerability in TNBC, whether linked to mitochondrial functions or not. Unlike Y705 phosphorylation, S727 is frequently enriched and constitutively active in the TNBC subtype, where it drives STAT3 dimerization, transcriptional activity, mitochondrial functions, and metastatic progression. Targeting STAT3 S727 phosphorylation—in combination with Y705 inhibition—consistently suppresses TNBC growth and dissemination with limited effects on other breast cancer subtypes, supporting S727 as a TNBC-selective therapeutic target.

An important open question is whether selective targeting of S727-dependent STAT3 functions may offer a favorable therapeutic window compared with p-Y705 or pan-STAT3 inhibition. Because canonical Y705-mediated transcription regulates a broad range of physiological processes including immune, survival and inflammatory responses, its systemic suppression may contribute to treatment-related toxicity [33,45,56,57]. In contrast, S727-dependent activities appear to be more closely linked to stress adaptation, metabolic rewiring and tumor-specific extranuclear functions, particularly in TNBC [19,25,101,102]. Although this distinction remains to be formally validated, selective modulation of the S727 axis may allow to preserve essential canonical STAT3 functions while interfering with tumor-promoting non-canonical signaling. The development of phosphorylation- and compartment-specific inhibitors will be essential to determine whether this conceptual therapeutic advantage can be translated into improved efficacy and tolerability.

**Table 3:** STAT3 inhibitors reported to interfere with S727 phosphorylation in TNBC, including the targeted domain, affected phosphorylated forms, and effects on both primary tumors and metastases. <, reduced; SUP, suppressed; LU, lung; BN, bone; LV, liver; N/A, not available.

Compound	Domain	p-S727	p-Y705	Exp. System	Primary Tumors	Metastases	Reference
7a	N/A	Yes	Yes	4T1	<	N/A	[101]
OPB-51602	SH2	Yes	Yes		N/A	N/A	[102]
SLSI-1216	SH2	Yes	Yes	MDA-MB-231	<	N/A	[37]
Niclosamide (NSA)	N/A	Yes	Yes	MDA-MB-231	<	<(LU, BN)	[42]
B19	SH2	Yes	Yes		N/A	N/A	[109]
RDp002	SH2	Yes	Yes	MDA-MB-468 4T1	<	<LU dissemination	[111]
Y002	SH2	Yes	Yes	MDA-MB-231	<	N/A	[112]
PSE2	SH2	Yes	Yes	MDA-MB-231	N/A	SUP (LU, LV)	[116]

## 8. Conclusions

STAT3 is a well-established oncogene whose pro-tumorigenic activity has classically been attributed to Y705-dependent transcriptional signaling [43]. However, accumulating experimental and clinical evidence now indicates that serine 727 phosphorylation represents a functionally independent regulatory axis that contributes to STAT3-driven tumor progression [65,66]. Elevated p-S727 STAT3 levels have been associated with aggressive disease features, metastatic dissemination, and poor clinical outcome in multiple cancer types, including PCa, ccRCC, and TNBC, often uncoupled from Y705 phosphorylation [30,42,87,88]. These findings highlight S727 phosphorylation as a critical determinant of non-canonical STAT3 functions that support tumor cell survival, metabolic adaptation, and invasiveness in TNBC [19,101,102]. Importantly, recent preclinical studies demonstrate that targeting p-S727 STAT3 can effectively suppress tumor growth and metastasis in NSCLC and TNBC models, positioning this form as a promising therapeutic vulnerability [37,42,101,106,111,112,116].

While Berkley and colleagues raised the possibility that inhibition of p-S727 STAT3 may contribute to treatment-related toxicity [58], the evidence cited derives from a dual STAT3 inhibitor (OPB-31121) and does not clearly isolate S727 inhibition as the causative factor. Toxicity observed in early-phase clinical studies is frequently influenced by multiple variables, including disease context, tumor burden, prior treatments, pharmacokinetics, and off-target effects. As such, currently available clinical data does not allow definitive attribution of toxicity specifically to STAT3 p-S727 inhibition. Indeed, as shown in Table 2, all compounds tested to date largely behave as dual inhibitors of both p-Y705 and p-S727 STAT3, and most are known to engage the SH2 domain, which is required for Y705 phosphorylation, dimerization, and canonical transcriptional activity [1,2,3,4,56,57,58]. Because the SH2 domain has not been directly implicated in mediating STAT3 p-S727-dependent extranuclear functions, reductions in p-S727 levels observed with SH2-directed agents are perhaps to be interpreted as indirect, potentially arising from upstream signaling rewiring, altered feedback loops, changes in phosphatase/kinase balance, or global effects on STAT3 stability and turnover. More studies are needed to shed light on the mechanisms implicated.

In addition to small-molecule inhibitors, emerging STAT3-targeting strategies include antisense oligonucleotides (ASOs) and proteolysis-targeting chimeras (PROTACs). To date, ASO-based approaches have primarily aimed at reducing total STAT3 expression and have been developed largely in the context of canonical Y705-dependent transcriptional activity in lymphoma and lung cancer, without assessing the predictably correlated S727 modulation [117,118]. Similarly, STAT3 PROTACs that effectively promote degradation of total STAT3 protein and impairs its transcriptional activity have been successfully applied in several different tumor types and likely affect both p-Y705 and p-S727 STAT3 levels, despite the latter rarely having been assessed [117,119,120,121,122]. Neither ASOs nor PROTACs have yet been investigated in TNBC [119,122]. Notably, Bai and colleagues reported a reduction in mitochondrial STAT3 pools upon treatment, confirming the idea that PROTAC-based strategies may also affect non-canonical STAT3 species [119]. However, in this study mitochondrial p-S727 was low or inconsistently detected at baseline depending on the cell type. Both ASOs and PROTACs are expected to present the same toxicity issues of other systemic STAT3 inhibition approaches in clinical settings. The development of compounds selectively modulating S727-dependent functions, rather than inducing complete STAT3 ablation, may represent a more refined and potentially better-tolerated therapeutic direction.

In addition, the compounds discussed here are heterogeneous in their dominant mode of action: some primarily reduce total STAT3 abundance (including mitochondrial pools), others chiefly impair mitochondrial function and/or subcellular distribution, and some mainly decrease the detectable p-S727 signal without clearly establishing direct engagement of the S727 regulatory axis. Therefore, to truly dissect the specific functions, therapeutic potential, and toxicity attributable to p-S727 STAT3, new compounds enabling selective and mechanistically validated modulation of p-S727 levels/activities are required.

Finally, while canonical and non-canonical STAT3 functions are mediated by different phosphorylated forms located at various sub-cellular compartments, in most instances both p-Y705 and p-S727 are known to contribute to tumor transformation and progression [3,12,15,21,25,26,30,59,65,89]. Because all alternative STAT3 species derive from a single pool of total STAT3, their balance is likely to represent a critical element of reciprocal cross-regulation (Figure 2), as also highlighted in several publications [69,75,79]. Although still largely inferential, this concept of competition for a shared protein pool provides a useful framework to interpret context-dependent STAT3 outputs.

From a translational perspective, implementation of p-S727 STAT3 as a clinical biomarker would require standardized and validated detection methods, most feasibly through validated phospho-specific IHC in TNBC tissue specimens. Parallel assessment of p-S727 and p-Y705 levels, together with evaluation of their subcellular distribution, could enable classification of tumors into distinct phosphorylation-defined categories (e.g., p-S727-high/p-Y705-low versus dual-high or dual-low), thereby identifying TNBC subsets preferentially dependent on S727-driven programs.

Integrating this information into biomarker-driven patient stratification and coupling it with phosphorylation-specific inhibitors may ultimately support more effective and better-tolerated STAT3-targeted precision therapeutic strategies.

## Data Availability

No new data was created or analyzed thus study. Data sharing is not applicable.

## Abbreviations

The following abbreviations are used in this manuscript:

ADT	Androgen Deprivation Therapy
ALM	Acral Lentiginous Melanoma
AR	Androgen Receptor
ASO	Antisense Oligonucleotides
BC	Breast Cancer
BM-MSC	Bone Marrow-derived Mesenchymal Stem Cell
BRET	Bioluminescence Resonance Energy Transfer
BTP	Benzothiophene
CCR1	CC motif Chemokine Receptor 1
CCRCC	Clear Cell Renal Cell Carcinoma
CIN	Cervical Intraepithelial Neoplasia
CLL	Chronic Lymphocytic Leukemia
CSC	Cancer Stem Cells
EC	Endometrial Cancer
EGFR	Epidermal Growth Factor Receptor
EMT	Epithelial–Mesenchymal Transition
ENZ	Enzalutamide
ER	Estrogen Receptor
ER	Endoplasmic Reticulum
ETC	Electron Transport Chain
FP	Fluorescence Polarization
GAS	Γ-Activated Sequence
GBM	Glioblastoma
GPCR	G Protein-Coupled Receptor
HAT	Histone Acetyltransferase
HER2	Human Epidermal Growth Factor Receptor 2
HIF-1α	Hypoxia-inducible factor 1α
IHC	Immunohistochemistry
IL-6	Interleukin 6
LC	Lung Cancer
LDHC	Lactate Dehydrogenase C
LIFR	Leukemia Inhibitory Factor Receptor
MAM	Mitochondria-Associated Membranes
MET	Mesenchymal–Epithelial Transition
MFS	Metastasis-Free Survival
MLS	Mitochondria Localization Sequence
MM	Multiple Myeloma
MMP	Matrix Metalloproteinases
MMTV	Mouse Mammary Tumor Virus
MPA	Medroxy Progesterone Acetate
NSA	Niclosamide
NSCLC	Non-Small Cell Lung Carcinoma
OS	Overall Survival
PCa	Prostate Cancer
PIAS3	Protein Inhibitor of Activated STAT 3
PR	Progesterone Receptor
PROTAC	Proteolysis-Targeting Chimera
PSE2	Pulchinenoside E2
PTM	Post Translational Modification
PTP	Protein Tyrosine Phosphatases
PV	Phospho-Valproic acid
RFS	Relapse-Free Survival
ROS	Reactive Oxygen Species
S727	Serine 727
S727A	Serine 727 Alanine
SERM	Selective Estrogen Receptor Modulator
SPTA	Sculponeatin A
SOCS	Suppressors Of Cytokines Signaling
SPR	Surface Plasmon Resonance
STAT3	Signal Transducer and Activator of Transcription 3
STAT3-C	Constitutively active Signal Transducer and Activator of Transcription 3
TKI	Tyrosine Kinase Inhibitor
TMZ	Temozolomide
TNBC	Triple-Negative Breast Cancer
VEGF	Vascular Endothelial Growth Factor
Y705	Tyrosine 705
Y705F	Tyrosine 705 Phenylalanine
